# *STXBP1*-Related Disorders: Clinical Presentation, Molecular Function, Treatment, and Future Directions

**DOI:** 10.3390/genes14122179

**Published:** 2023-12-05

**Authors:** Alexander Freibauer, Mikayla Wohlleben, Cyrus Boelman

**Affiliations:** 1 Division of Neurology, BC Children’s Hospital, Vancouver, BC V6H 3N1, Canada; alexander.freibauer@cw.bc.ca; 2 Faculty of Medicine, University of British Columbia, Vancouver, BC V6T 1Z4, Canada

**Keywords:** *STXBP1*, Munc18-1, epilepsy, genetics, developmental and epileptic encephalopathy, intellectual disability, SNARE protein

## Abstract

In recent years, the affordability and availability of genetic testing have led to its increased use in clinical care. The increased frequency of testing has led to *STXBP1* variants being identified as one of the more common variants associated with neurological disorders. In this review, we aim to summarize the common clinical phenotypes associated with *STXBP1* pathogenic variants, provide an overview of their known natural history, and discuss current research into the genotype to phenotype correlation. We will also provide an overview of the suspected normal function of the *STXBP1*-encoded Munc18-1 protein, animal models, and experimental techniques that have been developed to study its function and use this information to try to explain the diverse phenotypes associated with *STXBP1*-related disorders. Finally, we will explore current therapies for *STXBP1* disorders, including an overview of treatment goals for *STXBP1*-related disorders, a discussion of the current evidence for therapies, and future directions of personalized medications for *STXBP1*-related disorders.

## 1. Introduction

*STXBP1* is a gene located on chromosome 9q34.1 that encodes MUNC18-1, a presynaptic protein that is involved in a variety of cellular processes including exocytosis, neuronal viability, and cellular transport [1]. Pathogenic variants in *STXBP1* are associated with a wide range of clinical phenotypes, including global developmental delay (GDD)/intellectual disability (ID), Early Infantile Developmental and Epileptic Encephalopathy (EIEE), developmental epileptic encephalopathy (DEE), infantile epileptic spasms syndrome (IESS), and a clinical spectrum of movement disorders ranging from spastic tetraplegia to tremor, ataxia, and Parkinsonism-like features [2]. Due to the pleiotropic nature of *STXBP1*, it has become one of the most identified variants of concern, with a recent study of 150 children with EIEE identifying *STXBP1* variants in 6% of infantile onset epilepsy patients, and 10% of neonatal onset epileptic encephalopathy [3]. It has often been identified as one of the top five causative genes in a variety of genetic screening studies including infantile spasms [4,5,6,7], DEE [8,9,10], EIEE [11], and a combination of ID, GDD, and autism spectrum disorder [12]. Due to its importance within the cell and its role in clinical disease, *STXBP1* has become an area of research interest. A variety of cellular cultures, animal models, and in vitro studies have been developed to better understand its function and how abnormal variants can lead to clinical phenotypes. In addition, a variety of clinical and laboratory-based studies have been carried out to better understand optimal antiseizure medication options, as well as drug discovery to develop personalized therapies. In this narrative review summarizing a literature review through PubMed of papers mentioning “*STXBP1*” or “Munc18-1” from 2015 to 2023, we hope to provide a practical overview of *STXBP1* disorders, including clinical phenotypes suspicious of an underlying *STXBP1* variant, expected natural history of patients diagnosed with *STXBP1*, and will use an overview of the current scientific research to try to summarize the suspected underlying pathophysiology of *STXBP1*-related disorders to explore the current and future directions of management.

## 2. Clinical Phenotypes

Pathogenic variants in *STXBP1* were first identified in 2008 in five patients with Ohtahara syndrome (now known as EIEE) [13]. Over time, a variety of case reports were published identifying a wide variety of underlying phenotypes associated with STXBP1 variants. These included pathologies ranging from various types of DEEs (West syndrome [14], Lennox–Gastaut Syndrome [7], Dravet syndrome [15], and atypical rett syndrome [16]) to movement disorders [17] and intellectual disability [18,19]. In 2020, Xian and colleagues were able to identify 281 individuals previously reported in the literature and 253 individuals who were otherwise recruited through an international network of collaborators to provide a comprehensive overview of presentations associated with *STXBP1* variants. In their database using data standardized using Human Phenotype Ontology terminology, they identified that patients with *STXBP1* pathogenic variants most often presented with a combination of neurodevelopmental disorders, communication difficulties, gross motor delay, early-onset epileptic encephalopathy (including EIDEE and IESS), and movement disorders, with the remainder presenting with a variety of other DEEs [2].

### 2.1. Intellectual Disability

Intellectual disability was identified in 90% of patients with *STXBP1* variants over age 11, with the majority (64%) of these patients classified as having severe or profound ID, and only 2% with mild ID [2]. Intellectual disability has been observed in *STXBP1*-associated disorders both independently and comorbid with either epilepsy or movement disorders [17,19,20]. Concerning patients with ID and epilepsy, a natural history study looked at the developmental trajectories of patients with *STXBP1* and seizures, as measured by a specifically developed composite developmental score for patients with *STXBP1* [21]. They found that with a population of 48 patients with *STXBP1* pathogenic variants and seizures, the earlier onset of seizures was correlated with worse developmental outcomes, while age at seizure remission or duration of epilepsy did not affect outcomes [21]. In addition, 46% of these patients had developmental impairment and/or neurologic abnormalities before epilepsy onset [21]. A natural history study following patients into adulthood suggests that ID in patients with *STXBP1* is not static, with periods of developmental regression seen in 59% of 38 patients followed into adulthood [22]. These periods of regression were not reliably associated with an increase in seizure frequency.

### 2.2. Autism Spectrum Disorder

A smaller subset of patients have been noted to have autism-like features. The number of patients described as having autism or autistic-like features varies, with a range of 19–42% being reported in the literature [19,21,22,23]. Behavioral or psychiatric problems have also been reported, with a smaller subset of patients reported as having aggressive behaviors, hyperactivity, and awake bruxism [21,22,23,24,25]. A detailed neurodevelopmental assessment of 14 patients with *STXBP1*-related disorders noted that compared to other patients with ID, they often had more severe adaptive impairments, fine motor difficulties, and hyperactivity and often had worse receptive language and social impairments compared to more severe ID patients [26]. A unique feature found in patients with *STXBP1*-related disorders was a preservation of social motivation [26]. A disease concept model for *STXBP1* disorders was developed through interviewing caregivers and healthcare providers identified developmental delay as the most identified disease concept, with negative behavioral symptoms also described in 61% of mentions [27].

### 2.3. Communication Difficulties

Communication difficulties are also prominent in patients with *STXBP1* variants, with as many as 90% of patients having some degree of neurologic speech impairment [23]. A semantic similarity analysis using Human Phenotype Ontology-based phenotype descriptions from the whole-exome sequencing of 846 individuals with EE identified that “absent speech” is a gene-specific phenotype significantly associated with *STXBP1*-related encephalopathy [28]. This suggests that absent speech is a unique feature of patients with *STXBP1*-related disorders compared to other genetic epileptic encephalopathies. In patients identified with *STXBP1* variants, 38% of patients were identified as being non-verbal above the age of 11 [2], although in small cohorts, as many as 71% of adults with *STXBP1* were non-verbal [22]. Thalwitzer et al. noted that *STXBP1* patients with a history of epileptic spasms were three times less likely to be able to speak a single word, while other patients were able to communicate with age-appropriate or simple language. The earlier onset of epilepsy was also correlated with greater speech impairment compared to those with later seizure onset [23]. Communication difficulties are noted as having a significant impact on caregivers, with receptive communication difficulties having the second greatest impact behind only seizures [27].

### 2.4. Gross Motor Delay

Gross motor delay is common in patients with *STXBP1*-related disorders. In several retrospective natural history studies, about 50% of patients were able to independently walk [22,23], although ambulation was often acquired at a later developmental age [23]. Patients without comorbid epilepsy were more likely to have lower GMFCS scores (indicative of increased mobility) and four times more likely to ambulate unassisted. Conversely, patients with a prior history of epileptic spasms were less likely to walk unassisted [23]. In a cohort of patients with *STXBP1*-associated EE, about 46% of these patients present with axial hypotonia, with 23% having spastic/flaccid tetraplegia [21]. Functional mobility outcomes at an average of 24 years of age noted that 39% of patients were wheelchair-dependent, while only 50% were able to ambulate [22].

### 2.5. Epilepsy

Epilepsy is also a prominent feature of *STXBP1*-related disorders, with 75–89% of patients noted to have seizures [2,23]. Patients with *STXBP1* were reported to have a wide range of seizure types, with focal-onset (47%), generalized onset (43%), and epileptic spasms (42%) as the most common [2]. Most patients (89%) with seizures will have onset of seizures in the first year of life [2], with smaller cohorts noting a median onset at 1 month of life [21]. About 76% of patients will have remission of seizures in the first year of life [21], but even with periods of seizure freedom in childhood (described as many as 37% of patients with prolonged seizure freedom), 80% of patients have medically refractory seizures [22]. Most patients will have daily seizures, or clusters of seizures if the seizure type is spasms [21]. Status epilepticus is less common [2]. *STXBP1*-related disorders are noted to have a high frequency of associated epilepsy syndromes, including IESS and EIDEE.

IESS is found in 15% of patients with *STXBP1* pathogenic variants with seizures [2]. On the screening of all patients with IESS, *STXBP1* is one of the most common genes responsible, accounting for 4–17% of genetic etiologies [4,5,6]. Of note, *STXBP1* variants were found to be commonly associated with early-onset IESS (<3 months), and in a cohort of 86 patients with early-onset, IESS was found to be causative in 8% of cases [6].

EIDEE (a novel entity encompassing the syndromes formally known as Ohtahara syndrome and Early Myoclonic Encephalopathy) is also a common electroclinical syndrome associated with *STXBP1* pathogenic variants, accounting for 40% of *STXBP1* pathogenic variants [2]. Several screening studies to identify genetic etiologies of EIEE have noted *STXBP1* as a significant genetic etiology. A study assessing the utility of targeted gene panel sequencing in 150 EIEE patients identified *STXBP1* as causative in 6% of all cases [3]. In patients with neonatal onset EIEE, *STXBP1* variants were causative in 10% of cases [3]. A few other individual case reports report a heterogeneous group of EIDEE associated with *STXBP1* variants, including burst suppression [9,11,29] and dyskinesia [29]. *STXBP1*-associated EIDEE seizures have been associated with asymmetric tonic seizures, as well as sequential seizures (tonic, autonomic, clonic, and epileptic spasm) [30,31].

### 2.6. Movement Disorders

Patients with *STXBP1*-related disorders are also frequently seen to have a variety of movement disorders. Most seen are tremor and ataxia, which were identified in ~40% of individuals above the age of 11 [2]. Patients with ataxia are known to have non-progressive ataxia [32]. A study by Loussouarn et al. characterized this tremor in six patients. They found that it occurred mostly distally, predominantly during rest and with action, and was enhanced by emotions and concentration. When these patients underwent electrophysiological testing, it was noted that the patients all had distal or proximo-distal tremor-like rhythmic myoclonus during posture maintenance and action. This finding was corroborated by a study performing movement disorder video assessments, which noted a jerky tremor in 5/17 patients and convincing features for myoclonus in 2 of these patients. Due to the diffuse nature of the myoclonus, they postulated that the gait ataxia normally seen with *STXBP1*-related disorders may be due to lower-limb myoclonus, although this has not been tested yet [32]. Motor stereotypies have also been identified in 31–63% of patients, mainly involving hands, but also seen in oral and stereotypies involving the head [21,22]. Movement disorders in *STXBP1*-related disorders occasionally are only comorbid with ID. This had previously been described as ataxia–tremor–retardation syndrome without epilepsy [17,24]. A variety of other movement disorders have been identified as well, including hypomimia, bradykinesia, dystonia, dyskinesia, and choreoathetosis [21,22,24,32,33].

### 2.7. Other Phenotypes/Systemic Symptoms

*STXBP1*-related disorders present with a wide variety of other phenotypes consistent with DEE. Most described are cases of atypical Rett syndrome (2%), which were described as cases with noted hypotonia, motor hand stereotypies, and epilepsy [2,16]. These cases appear to be consistent with the classic findings generally associated with *STXBP1* variants and may represent a misclassification as atypical Rett syndrome. Other electroclinical syndromes associated with STXBP1 variants include Lennox–Gastaut syndrome [7,34] and Dravet syndrome [15]. Summarized electroclinical syndromes are described in detail in Table 1.

*STXBP1*-related disorders have been noted to have a variety of other systemic comorbidities. In about 20% of patients, they have difficulty with sleep initiation/maintenance [21,22]. In adult patients with *STXBP1*, about 50% had GI-related issues, including constipation, difficulties with feeding, and GERD [22].

*STXBP1* brain imaging in general has been noted to be normal in about 50% of patients, but what also can be seen is mild cortical atrophy, a thin corpus callosum, and hypo/delayed myelination, plus rare cases of focal cortical dysplasia have been reported [19,21,37].

### 2.8. Natural History

The natural history of patients with *STXBP1*-related disorders remains an understudied aspect of *STXBP1*-related disorders. As described above, patients with STXBP1-related disorders often have early-onset epilepsy, intellectual disability, and/or static movement disorder. As they age, they are found to have fluctuating periods of seizure remission, but overall will continue to have medically refractory epilepsy throughout their lifespan [22]. In addition, they are also noted to have periods of developmental regression independently of seizure burden [22]. A recent study of *STXBP1*-related patients into adulthood found that 50% lived in residential care and the majority were completely dependent on caregivers for most activities of daily living [22]. As discussed previously, patients with early-onset seizures were found to have lower developmental scores, as established by Balagura et al., and those with IESS were more likely to have higher GMFCS scores and communication difficulties [23].

It is also likely that due to genetic testing in specific clinical scenarios such as for intellectual disability and epilepsy, we may not be capturing the breadth of phenotypes associated with *STXBP1* variants. Continued genetic testing of a broader range of phenotypes in the adult and pediatric population will likely identify milder phenotypes associated with *STXBP1* that may present at later stages of life that have not been identified at this time. Diligence in the identification and clinical monitoring of this population is essential to a more comprehensive understanding of *STXBP1*-related disorders.

## 3. *STXBP1* and Munc18-1 Structure and Function

*STXBP1* is a gene located on chromosome 9q34.11 that encodes Munc18-1, also known as syntaxin-binding protein 1 [1]. *STXBP1* consists of 20 exons encoding 594 amino acids arranged into four domains (1, 2, 3a, and 3b) [2,38]. STXBP1 has been noted to have two splice variants, *STXBP1* long splice variant (STXBP1L) of 603 amino acids and *STXBP1* short splice variant (STXBP1S) of 594 amino acids which differ in their C-terminal amino acid sequences [39]. STXBP1S is expressed predominantly in early embryonic stages, while STXBP1L only tends to increase in expression after birth [39].

### 3.1. Genotype/Phenotype Correlation

A wide variety of genotypes have been observed in patients with STXBP1-variant-associated disorders. The mechanism of action these variants have predominantly shown is one which causes haploinsuffiency or exerts a dominant negative effect, but rare cases have shown a homozygous gain of function effect [2,34]. Of the 534 individuals identified by Xian et al., the nature of germline variants included missense variants (*n* = 255), protein-truncating variants (*n* = 119), splice site variants (*n* = 79), whole or partial gene deletions (*n* = 33), frameshift variants (*n* = 30), duplications (*n* = 5), and in-frame deletions (*n* = 9). These variants were dispersed throughout the protein, occurring with greatest frequency compared to population variants in between domain 2 and 3a and within domain 2 near the C-terminus. Of these variants, there were 54 recurrent variants accounting for 32% of cases, most commonly affecting Arginine residues. The three genomic hotspots of greatest frequency were p. Arg406Cys/His (*n* = 40) located in domain 3b, p. Arg292Cys/His/Leu/Pro (*n* = 30) located in domain 3a, and p. Arg551Cys/Gly/His/Leu (*n* = 24) located in domain 2 [2]. In a comprehensive context analysis of 136 de novo/rare mutation (SNV/Indels) sites in this gene, 27% of all SNV mutations occurred within five base pairs upstream or downstream of a ‘GTA’ motif [40]. Somatic variants have also been identified in a minority of cases, with a study identifying a somatic mutation in *STXBP1* in excised focal cortical dysplasia type1a tissue [37]. A parental somatic mosaicism may also contribute to suspected ‘de novo’ mutations as at least one parent of a child with *STXBP1*-related disease was noted to have somatic mosaicism [41].

By using semantic similarity analysis, Xian et al. established genotype/phenotype correlations for their patient population. They found that protein truncating and deletion variants had similar phenotypic similarity, often presenting with IESS and ataxia, while missense variants were more likely to have EIEE/DEE [2]. Looking at the common recurrent variants, they were able to hypothesize a likely genotype/phenotype correlation for each genomic hotspot, although no significant phenotypic variability was observed. p. Arg406Cys/His variants were found to be associated with EEG with burst suppression, spastic tetraplegia, and inability to walk. p. Arg292Cys/His/Leu/Pro variants were more likely to have head tremor, focal seizures with impaired awareness, and abnormalities of their ventricles. p. Arg551Cys/Gly/His/Leu variants were associated with developmental regression, EEG with slowing and generalized seizures, and were three times less likely to have infantile spasms [2]. Splice site variants in patients with *STXBP1*-related disorders were also reviewed to look for a potential genotype/phenotype correlation. Wang et al. [42] looked at 54 canonical splice variants from Clin Var and used splice AI to predict potential splicing changes. Canonical variants were found to cause frameshift or deletion effects, presenting with a phenotype seen in PTV/del variants by Xian et al.

To foster a better understanding of how *STXBP1* variants are thought to cause disease, we will first go over their suspected normal function within the cell. Munc18-1 has broad effects within the cell, impacting exocytosis, neuronal viability, endocytosis, and cellular transport. In exocytosis, Munc18-1 has been found to play a role in vesicle release through the SNARE complex, as well as the release of dense core vesicles (DCV) [1,43,44,45].

### 3.2. STXBP1 Physiologic Function

SNAP25 receptor (SNARE) proteins are a heterogenous family of proteins that with key regulators are important in synaptic vesicle exocytosis and synaptic transmission [46]. The SNARE complex consists of synaptobrevin-2 (VAMP2), a vesicular protein that binds to a complex of membrane proteins syntaxin1-A (STX1A) and SNAP25. The assembly of SNARE machinery is carefully arranged by Munc18-1 and Munc13-1 [47]. The SNARE complex subsequently is responsible for vesicle fusion at the membrane in response to calcium influx binding to the calcium sensor protein synaptotagmin [1,46].

Munc18-1’s role in the SNARE complex is to regulate assembly. Munc18-1 at the baseline binds to syntaxin-1, preventing syntaxin-1 from forming a SNARE complex. Munc18-1 is then thought to interact with Munc13-1 to remove the inhibition of STX1A, permitting STX1A to bind to VAMP2. Munc18-1 then acts as a template by which the SNARE complex can form on its surface [1]. In addition, Munc18-1 is found to overcome the secondary inhibition of the SNARE complex by the aSNAP protein through the competitive binding of STX1A [44]. Munc18-1 has been found to be essential for neurotransmitter transmission, with knock-out mice generated via homologous recombination found to have no neurotransmission activity recorded, and heterozygous mice showing the slower release of synaptic vesicles [46,48].

Recent experiments have also suggested that Munc18-1 could play a role in DCV release. DCV release is not as well understood as synaptic vesicle release, but research by Puntman et al. showed that in Munc18-1 null mice, neuropeptide release was abolished. They were also able to show that by expressing Munc18-1 using the Cre-lox construct in Munc18-1 null neurons, they were able to rescue DCV release. In addition, they noted that in Munc18-1 heterozygous mice, DCV release was similarly impaired. These findings implied that Munc18-1 is essential for the secretion of DCV, which is often required for the release of neurotrophic factors and axon guidance molecules [43]. This is supported by findings outside the brain, where it was found that Munc18-1 plays a role in facilitating the release of insulin via DCV [49].

Munc18-1 also plays a role in neuronal viability. In cultured mouse central nervous system neurons, a lack of Munc18-1 led to massive cell death before synaptogenesis [50,51]. It is thought that this role of Munc18-1 is separate from its role in synaptic function as neuronal viability can be rescued through the expression of non-neuronal paralogs but does not restore synaptic transmission [50]. In Munc18-1 null mutant brains, neuronal cell death also appears to follow a developmental pattern, starting in lower brain regions that mature first and gradually move to cortical regions [52]. The transcriptomic and proteomic profiling of hippocampal cells in *STXBP1* knock-out mice generated by homologous recombination was performed to better understand how it impacts neuronal viability. The analysis of transcripts showed a downregulation of transcripts related to neuronal function, while proteosome analysis indicated the dysregulation of proteins involved in synaptic transmission and neuronal development [52]. Although no specific pathway was identified, a lack of *STXBP1* expression appears to have a broad impact on neuronal function and development.

**Figure 1:**
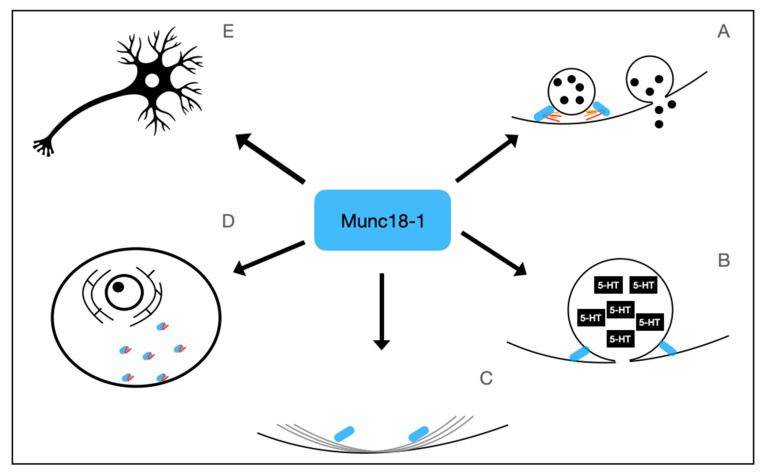
Physiological Functions of Munc18-1, the protein encoded by *STXBP1.* Munc18-1 has numerous physiologic functions. It plays an important role in regulating SNARE complex formation (**A**), Dense-core vesicle release (**B**), regulation of F-Actin network (**C**), anterograde and retrograde transport of vesicles and syntaxin 1A (**D**), and is involved in neuronal viability (**E**).

Munc18-1’s role in neuronal viability may also be due to its impact on intracellular transport and endocytosis. Munc18-1 regulates the submembrane F-actin network, an important network facilitating vesicle transport [53]. Munc18-1 expressed by STXBP1S binds to Myosin Va and has been shown to help facilitate the trafficking of Syntaxin 1A to the presynaptic terminal [39]. In addition, Munc18-1 has been found to play a role in the retrograde endosomal transport pathway, with Munc18-1 null cells having a significantly decreased retrograde uptake of TrkB receptors compared to the controls [52]. In addition, *STXBP1* null neurons generated by homologous recombination in mice were found to have a significant reduction in dynamin protein and transcript levels, a protein that is required for the specific pathways of endocytosis [54]. The physiologic function of Munc18-1 is summarized in Figure 1.

## 4. Pathophysiology of *STXBP1*-Related Disorders

*STXBP1*-related disorders are thought to primarily be secondary to the haploinsufficiency of Munc18-1 [1,55], but some functional data may suggest a dominant-negative mechanism for some variants, including p.Arg406His [2], and two patients with homozygous missense mutation in p.Leu446Phe have been shown to have a gain of function mechanism [34]. How STXBP1 haploinsufficiency can lead to the pleiotropy seen in *STXBP1*-related disorders has not yet been fully elucidated.

**Figure 2:**
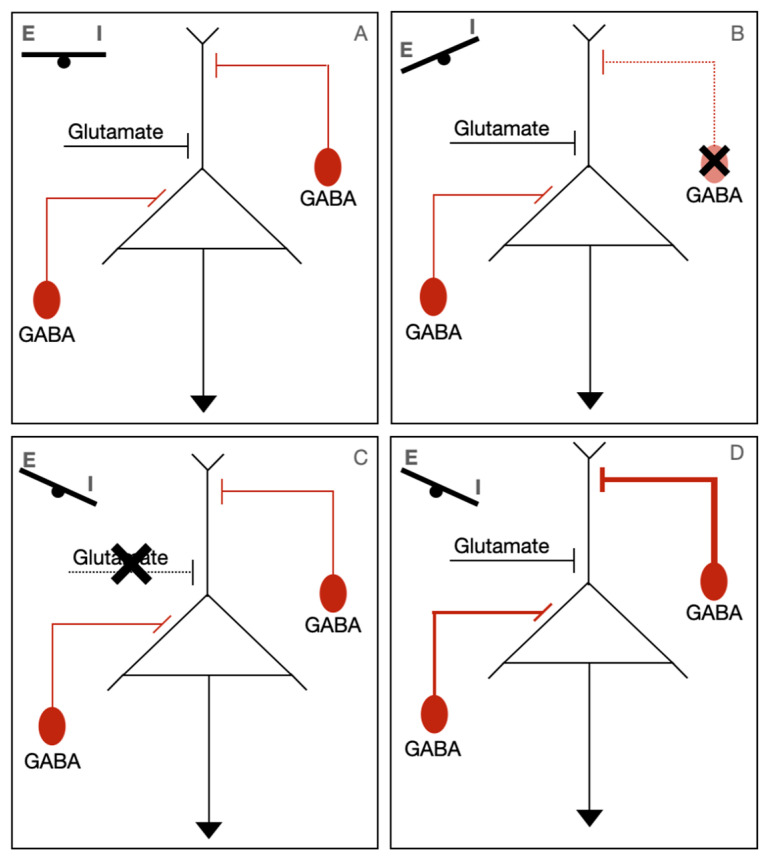
Potential pathophysiological mechanisms of STXBP1-related disorders. Normally, there is a balance of excitatory (Glutamate) and inhibitory (GABA) inputs (**A**). In STXBP1-related disorders, it is thought that synaptic dysfunction upsets the balance of excitation/inhibition (E/I). The three proposed mechanisms of how there can be a disruption of this balance are either localized GABA associated neuron dysfunction (**B**), impaired excitatory transmission (**C**), or a breakdown in synchrony of GABAergic/glutamatergic neurons (**D**).

One theory presented by Verhage et al. suggests that *STXBP1* disorders are part of a “SNAREopathy”, in which impaired synapse function can lead to intellectual disability, seizures, and ASD. The basis for this theory lies in the finding that all these phenotypes have been linked to a dysregulation of excitation/inhibition (E/I) balance in brain circuits. They proposed three mechanisms of how synaptic impairment can cause this imbalance. First is that haploinsufficiency affects the GABAergic interneuron directly, leading to the failure of inhibition. Second, impaired excitatory transmission may lead to the impaired recruitment of interneurons, leading to impaired synchronicity. Thirdly, a breakdown in the synchronicity/inconsistent activation of interneurons could potentially lead to prolonged or ill-defined summation windows, which have been associated with ID. These findings are summarized in Figure 2. Circuit dysfunction was also theorized to lead to movement abnormalities due to difficulty with coordinating complex movements.

This theory has been supported through several recent studies. First, a study looking at the dynamics of routine EEG in STXBP1-related disorder patients noted that patients with *STXBP1* had inhibition-dominated networks compared to healthy controls as measured by a functional excitation/inhibition ratio [56]. This suggests that patients with *STXBP1* have excitation/inhibition dysregulation, which has been shown in other patients with ID/epilepsy. Secondly, in a novel in vitro human assay using a living organotypic cultures of human subplate regions that was induced to have *STXBP1* haploinsufficiency, it was noted that there was a significant downregulation of glutamatergic synapses, with a compensatory increase in GABAergic synapse [57]. The transcriptome analysis of the prefrontal cortex of base-edited cynomolgus monkeys with an *STXBP1* variant showed a overall reduction in the ratio of interneurons compared to wild-type monkeys, as well as a decrease in a subpopulation of excitatory neurons [58]. The selective degeneration of specific excitatory and inhibitory neurons provides evidence for how impacts on neuronal viability could affect cortical synchronicity. This functional imbalance was also observed in Zebrafish carrying an LOF deletion in an *STXBP1* paralog. Zebrafish imaged with in vivo fast confocal calcium imaging noted that in the *STXBP1* LOF zebrafish, there was more prominent and larger-scale neuronal cascade activity, suggestive of circuit dysfunction [59]. Overall, these findings are supportive of *STXBP1* haploinsufficiency, leading to increased dyssynchrony in developing cortical circuits.

Munc18-1’s other physiologic roles may also help explain other aspects of the *STXBP1*-related disorder phenotype. Munc18-1’s role in DCV release may lead to a variety of currently undefined downstream effects due to an inability to properly release neuropeptides and neuromodulators [1].

## 5. Treatment

Based on our current understanding of *STXBP1*-related disorders, several targeted therapies have been developed to help treat this patient population. Levetiracetam (LEV), an antiseizure medication whose mechanism of action targets the synaptic vesicle protein SV2A, has been found to have some benefit in patients with *STXBP1* disorders [60]. A comparison of 26 patients initiated on LEV compared to 10 patients on other antiseizure medications showed a significantly greater number of patients with >50% seizure reduction at 6 months (88% vs. 50%). Although LEV did lead to a reduction in seizure frequency, there was no observed difference in achieving seizure freedom [60]. A few case reports anecdotally state that levetiracetam has benefits as well [42,61]. In contrast, a larger-scale analysis of patients with *STXBP1* by Xian et al. found that LEV had an odds ratio <1 in reducing seizure frequency and maintaining seizure freedom, despite being one of the most prescribed medications. This suggests that LEV may not be as effective as once thought for seizure control in patients with *STXBP1*-related disorders.

Novel targeted therapies in *STXBP1*-related disorders now include the development of chaperone proteins [62]. Missense mutations of Munc18-1 are thought to lead to destabilization of the mutant protein, leading to a haploinsufficiency, resulting in the abnormal phenotype. The goal of chaperone proteins would be to stabilize the abnormal protein to restore the normal function of Munc18-1. In vitro studies with mice neurons were able to identify two compounds that were able to stabilize the abnormal protein and restore synaptic function [63]. Chaperones are an interesting possible future personalized treatment option for patients with *STXBP1*-related disorders.

Another treatment option that has been considered is to trial medications that interact with serotonin receptors. The screening of various antiseizure medications in a zebrafish model of *STXBP1*-related disorders identified Clemizole and Trazodone as medications that decreased ictal events [64]. These findings suggest that medications with serotonin-receptor-binding affinities could be an effective antiseizure medication option, although further research is needed.

With respect to the general management of seizures in patients with *STXBP1*-related disorders, Xian et al. showed that medications that have the most benefit in treating seizures are those used for the management of IESS (ACTH and Prednisolone), although this may be due to the natural history of IESS, leading to the remission of seizures within the first year of life. The most effective treatment otherwise identified was the ketogenic diet or clobazam in reducing seizure frequency or maintaining seizure freedom [2,65]. VNS has also been effective to improve psychomotor function as well in a smaller case report [66]. Epilepsy surgery should always be considered in *STXBP1* patients as well. Several cases with focal epilepsy have been identified with focal cortical dysplasia due to both germline and somatic genetic mutations in *STXBP1* mutations, with good seizure control upon lesionectomy [37,67,68]. Epilepsy surgery evaluation should always be considered in patients with focal medically refractory epilepsy even if there is a suspected genetic etiology.

## 6. Future Directions

With recent developments in targeted treatments for *STXBP1*, further work needs to be done to better understand the goals of treatment for patients with *STXBP1*-related disorders. Currently, reduction in seizure frequency is often used as a marker of treatment success, but in *STXBP1*-related disorders, patients often have comorbid intellectual disability and movement disorders that significantly impact their quality of life. A recent disease model of *STXBP1*-related disorders noted that developmental delay, receptive communication, and behaviors have greater impacts on individual patients and caregivers than seizures [27]. Although initial natural history studies have been conducted to explore the typical progression of patients with *STXBP1*-related disorders using a GMFCS scale and expressive language score [23], as well as a designed *STXBP1* development score [21], further studies are needed to further characterize a developmental and functional baseline to assess the benefit of future therapies. The use of already validated scales (such as the Vineland Adaptive Behavior Scale or Social Responsiveness Scale) or the validation of novel scales such as the *STXBP1* development score may be useful ways to accomplish this goal.

Several animal models and in vitro assays have been developed to help facilitate future drug discovery. In recent years, a zebrafish animal model for *STXBP1*-related disorders has been developed [69,70]. Already these models have been used to screen antiseizure medications [64] and have been used to visualize in vivo network dynamics [59]. This may function to easily screen medications, as well as monitor changes in network dynamics. An animal model of *STXBP1*-related disorders has also been developed in cynomolgus monkeys, which may provide an opportunity to provide more in-depth behavioral testing in the testing of pharmacological agents [58]. In addition, the development of a human in vitro model of early cortical development may be a useful medium to assess the potential therapeutic benefits of chaperones in human cortical tissue [57].

In addition, there is potential for the development of new categories of treatments. Considering that most *STXBP1*-related disorders are due to haploinsufficiency, there is the possibility of gene therapies to upregulate the normal copy of *STXBP1*. Research has shown that physiologic microRNA can target the *STXBP1* transcript and significantly decrease transcription [71]. Therapies to target microRNA may be beneficial in increasing the levels of *STXBP1*-encoded Munc18-1 protein in the cell. The development of gene therapies treating haploinsufficiency-induced conditions such as Dravet syndrome provides hope that similar therapies can be developed for *STXBP1*-related disorders in the future.

Currently, there are a few clinical trials that are ongoing for therapeutic treatments for *STXBP1*-related disorders that have yet to be published. These include small single-center studies using the previously FDA-approved medication 4-phenybutyrate in patients with *STXBP1*-related disorders compared to patients with *SLC6A1* [72], and a small pilot non-blinded study testing the effectiveness of fenfluramine in several DEEs, including patients with *STXBP1*-related disorders [73]. There is hope that these trials will have a future benefit for patients with *STXBP1*-related disorders.

## 7. Conclusions

*STXBP1*-related disorders are typically characterized by a combination of intellectual disability, early-onset epileptic encephalopathies, gross motor dysfunction, movement disorders, and communication difficulties. Clinical phenotypes that should raise the suspicion of *STXBP1*-related disorders include the onset of infantile spasms < 3 months of age, intellectual disability with ataxia and tremor, as well as patients with absent speech with early-onset epilepsy and intellectual disability. The natural history of *STXBP1*-related disorders is suggestive of life-long medically refractory seizures, with patients with early-onset seizures having worse developmental outcomes. Most patients in adulthood are dependent on caregivers. Genotype–phenotype correlation is not definite, but variants that lead to protein truncation/deletion are more likely to be associated with ataxia and IESS. *STXBP1*-related disorders are mostly due to haploinsufficiency. Chaperone-based therapies and serotonin-affecting agents are possible future targeted therapies for *STXBP1*-related disorders, but further research into patient’s natural history is required to fully understand the potential treatment benefit beyond seizure control. For the management of seizures, ACTH and prednisolone are effective to treat IESS, while the ketogenic diet and clobazam are found to be the next most effective in managing seizures. The increased use of genetic testing has been crucial for our understanding of *STXBP1*-related disorders, and its broader use in years to come will help us better understand the phenotypes of this disorder to provide better prognostication and help in the development of targeted therapies.

## Figures and Tables

**Table 1:** Common epilepsy syndromes associated with *STXBP1*-related disorders [35,36].

Syndrome	Seizure Onset	Seizure Types	Neurodevelopmental Symptoms	Proportion of STXBP1 Patients
Early Infantile Developmental Epileptic Encephalopathy	0–3 months of age	Tonic and/or myoclonic seizures	Developmental impairment is prior to or shortly after onset of seizures	40%
Infantile Epileptic Spasm Syndrome	1–24 months of age	Flexor, extensor or mixed epileptic spasms, which often occur in clusters	Developmental slowing after spasms onset, but may be absent early in course	14%
Atypical Rett Syndrome	N/A	N/A	A period of regression followed by recovery or stabilization with 2/4 of main criteria (loss of acquired purposeful hand skills, loss of acquired spoken language, Gait abnormalities, stereotypic hand movements) with 5/11 of supportive criteria	2.6%
Lennox-Gastaut Syndrome	Seizure onset prior to 18 years. (Often progress from IESS or severe infantile epilepsy syndrome)	Tonic seizures with at least 1 other seizure type (atypical absence, atonic, myoclonic, focal impaired awareness, generalized tonic-clonic, epileptic spasms)	Often have developmental slowing, plateauing or regression with moderate to severe ID in >90% of patients	<1%
Dravet Syndrome	6–15 months of age	Focal seizures with impaired awareness, Absence seizures, Myoclonic seizures, Atonic seizure	Often normal development followed by developmental plateauing	<1%

## Data Availability

Data sharing not applicable. No new data were created or analyzed in this study. Data sharing is not applicable to this article.
